# The Transactions of the Provincial Medical and Surgical Association

**Published:** 1840-01

**Authors:** 


### Art. VIII.

*The Transactions of the Provincial Medical and Surgical Association.* Vol. VII.—*London and Worcester*, 1839. 8vo, pp. 574.

This volume, like its predecessors, opens with a report of the proceedings at the previous meeting of the Association; but as we have already had occasion to notice these, we shall proceed at once to give some account of its literary contents. The first article is Dr. Malden's retrospective address. Although it is evident that the professional avocations of that gentleman have prevented him from engaging in the laborious and extensive research requisite to produce a full abstract of the improvements and other novelties—the latter, as usual, materially outnumber the former—of the previous year, especially of those originating in foreign countries, yet his elegant oration will be read with no common interest. The brief enquiry into our recent national contributions to medical science is judiciously and candidly conducted, and exhibits Dr. Malden, in the light of an experienced practitioner, closely scrutinizing the views of others, by the aid of his own lengthened clinical acquaintance with disease. As a literary composition, Dr. Malden's essay possesses merit of a very high order, and may be triumphantly appealed to in refutation of those who affirm that the character of the studies and pursuits of medical men unfits them to become graceful and elegant writers.

I. The Transactions are, as usual, remarkable for the importance of their topographical papers. One of the corresponding members of the society, Professor Otto, of Copenhagen, has enriched the present volume with an elaborate account of the medical peculiarities of that capital. Here we have an essay exuberant with matter, all of which is admirably arranged, while each particular topic is treated of with the degree of fullness and precision due to its relative importance. Dr. Otto commences by passing in review the situation of Copenhagen; its buildings; the modes of ventilation and warming practised in private houses; the climate of the town and surrounding districts; the physical and mental constitution of its inhabitants; their public and private amusements; their habitual food and manner of living; and, finally, the means employed by the authorities for the preservation of the general health. The examination of these topics constitutes the natural exordium of such a paper as the present, as it embraces all those agencies which past experience has taught us to regard, as capable of influencing the character of disease, and the efficiency of remedies in a particular region. We might extract, to a large amount, from this portion of the essay, with the certainty of engaging the reader's attention, but must content ourselves with recommending it to his perusal. The regulations adopted by the government for limiting the sale of poisons to proper persons, for preventing the consumption of spoiled food, and the use of noxious or poisonous colouring matters in the confection of sweatmeats, &c., must, if actually put into practice, and not, as is too often the case elsewhere, merely nominally prescribed, exercise a very material influence on the prevention of the evils they are meant to check. That the enlightenment of Danish medical policy is not, however, without its limits, clearly appears from the fact, that of the five members of the Royal Board of Quarantine, not one belongs to the medical profession. The evils likely to arise from this arrangement are in a great measure averted, according to Dr. Otto, by correspondence between the Board and the Royal College of Health; we doubt the success of the remedy, and may, with fairness, remind the Professor of the old adage, "prevention is better than cure." The notions prevalent in Denmark, on the subject of contagion, are, it would seem, somewhat antiquated, and requiring modification, quite as much as the organization of the Board of Quarantine. That "Copenhagen is much exposed to the importation of the *yellow fever*" is surely an assertion at which, although it is qualified by certain provisoes, we may, at the present day, be permitted to marvel.

Dr. Otto considers the diseases of Copenhagen under the three heads of *epidemic*, *endemic*, and *contingent*. There is nothing particularly worthy of notice related respecting diseases of the first class, with the exception of variola. After briefly alluding to various outbreaks of this disease, which have occurred at Copenhagen since 1528, the writer proceeds to describe the observations made during an epidemic of the disease, which has lasted from May, 1832, up to the present time. Of 1045 patients admitted into the hospital during this period, 44 died: 147 of these individuals, who had not been vaccinated, furnished 34 of the deaths; while 10 only of the remaining 898 persons, all of whom had been vaccinated, perished. The general inferences, drawn from the observation of the epidemic, are expressed by Dr. Otto, in a number of propositions, of which the following strike us as most important: "The prophylactic power of the vaccine gradually decreases, and after the course of some years, ceases entirely, with the greatest part of the vaccinated. The character of the cicatrices, after vaccination, is in no proportion to the greater or less modification of the smallpox." We shall shortly have an opportunity of examining the validity of these propositions. We shall now content ourselves by stating that the first is anything but proved.

Belladonna is no longer, we are informed, regarded as a prophylactic against scarlatina. If Dr. Otto's estimate of the intellectual character of his countrymen—namely, that they possess more judgment than imagination—were strictly correct, they would not have been so long in arriving at their present conclusion; they would have seen through the hollowness of the evidence on which the alleged preventive powers of the drug rested, and quietly added the tale to the host of fallacies originating with dreamy speculators.

The endemic diseases of this insular city are, ague, catarrh, rheumatism, gout, hemorrhoids, scrofula, tubercular consumption and hydrocephalus, autumnal cholera, psora, and a syphiloid affection resembling the Scotch *sibbens;* stone, dysentery, and scurvy, are represented as of rare occurrence.

Catarrh, manifesting itself by cough and coryza, is stated to lay the frequent foundation of phthisis and other intractable diseases. We are persuaded Dr. Otto is too well acquainted with the rigorous demonstration required, in more southern latitudes than his own, of such important practical facts as this, to feel irritated at our unwillingness to credit the reality of the assumed correlation of bronchitis and tubercle.

So long as Dr. Otto withholds the proofs of his assertion, we shall continue to believe, that the laws discovered to regulate the relations of those diseases, in other climates, hold good in the capital of Denmark. Two tables are given, from which it appears that tubercle produces one eighth of the total mortality and one thirtieth of the total sickness of Copenhagen.

Sixty deaths occurred from hydrocephalus in 1835, and sixty-two in 1836, showing the extreme frequency of the disease. Our author is disposed, with many of his colleagues, to consider this affection of scrofulous nature, from its especially attacking children of that constitution. He appears to be unaware of the much stronger reason afforded by the pathological anatomy of the disease, for regarding it in the majority of instances as of tuberculous nature. The reader need not be informed that we refer to the presence of tubercles in the pia mater of hydrocephalic patients, a condition forming the anatomical character of the disease described by our countryman, Dr. Hennis Green, under the title of *granular meningitis.*

We pass with our author to the category of contingent affections. Genuine sthenic inflammations appear, on the whole, to be somewhat rare in Copenhagen, scarcely one out of twenty inflammatory disorders being of that type. Those met with are stated to be "rheumatic, nervous, or gastric complications, and varying according to the different seasons and the state of the weather." Of what these conditions are complications we are not informed. Venesection is consequently not carried to any great extent; in cases of pneumonia, for example, one or two bleedings of twelve ounces each are usually found to be sufficient; tartar emetic has of late been used with great advantage after venesection. Calomel and opium are often prescribed, particularly in inflammations of the lungs and liver; "though not so much and so indiscriminately as in England:" for this last the Danes have good reason to thank their stars.

The treatment of typhoid fever pursued by Professor Bang is described at some length, and emphatically lauded: "such is its happy result, that of three thousand patients, only three hundred and seventy died." In other words, one of every eight cases terminated fatally. Now of one hundred typhoid patients treated by M. Louis, simply with emollient ptisans, seltzer water, and linseed enemata—a mode of treatment scarcely differing from pure expectancy—nine only died, that is, one in eleven. And yet, as we can ourselves testify, his cases were frequently of the severest kind, accompanied with intestinal hemorrhage to a considerable amount, the formation of large eschars, &c. The natural course and mortality of disease should at least be ascertained before we pronounce panegyrics on our treatment.

We have so recently laid before our readers a full analysis of Dr. Guldberg's remarkable work on Delirium Tremens,* of which the materials were collected at Copenhagen, that scarcely any novel matter referring thereto can be gleaned from the present essay. The relative frequency of the disease is, however, calculated through a longer term of years than in Dr. Guldberg's volume. "From the year 1826 to 1836

* See British and Foreign Medical Review. Vol. VI., p. 320.

inclusive, of 23,765 patients admitted into Frederick's Hospital, 990 (1 in 24) had delirium tremens,—a number which must astonish the reader, the more when he reflects that this has occurred in one hospital only, and an equal and perhaps still greater number must have been treated in the general hospital, as well as in the military and naval hospital and in private practice." Dr. Otto's patriotism no doubt prevented him from inserting delirium tremens in his list of *endemic* diseases: it is plain, however, that Shakspeare was right (he did not often err) in setting down " your Dane " as a great drinker.

The majority of Danish practitioners still continue to treat syphilis with mercurial preparations, especially with calomel; when symptoms of ptyalism appear the medicine is discontinued, but resumed when they have been removed. Twelve years ago, however, Dr. Otto, on his return from a visit to this island, turned his attention to the non-mercurial treatment of the disease; several of his brethren have since made trial of the plan, and the following is the statement given of their joint experience of it: " The results which were published proved the value of the method even beyond expectation; so that I now never use mercury against syphilis, effecting a complete cure of all its forms within a much shorter time than was formerly done by mercury, and without seeing secondary symptoms arise afterwards. Professor Wendt, Dr. Müller, and Dr. Svitzer have been equally successful with this mode of treatment; though they think that there are forms of syphilis which require mercury, and consequently now and then employ it. I frankly confess that I have not seen such cases."

The subjoined figures will give the reader some idea of the movement of the population of the kingdom of Denmark during one year, 1835:

| Population. | Marriages. | Births. | Deaths. |
|---|---|---|---|
| 1,234,000. | 10,088. | 41,052. | 30,170. |

Proportion of girls to boys as 1000 : 1053.
.... of do. to do. born dead as 1000 : 1531.
.... of illegitimate to legitimate births as 1 : 8.
.... of deaths to whole population as 1 : 41.*
Number of deaths by suicide 192.

The proportion of illegitimate births would appear to be greater than in any other country; from 1831 to 1835 there were 197,300 births, and of these 18,688, or 9 per cent., were illegitimate.

III. *On the Medical Topography of Swansea.* By Mr. J. W. Gutch.

IV. *On the Medical Topography of Exeter.* By Dr. T. Shapter.—We postpone, for the present, our intended notice of these papers: the latter is indeed still uncompleted.

V. *On a Peculiar Affection of the Uvula.* By Mr. E. Thompson, of Whitehaven.—Mr. Thompson is of opinion, that in cases of simple inflammation of the throat terminating suddenly by death, the fatal event is caused by asphyxia, from enlargement of the uvula. " The affection

* In England it is 1 in 49. See Farr's letter to the Registrar-General.

consists in the elongation of the mucous coat of the uvula, by the gradual effusion of lymph, to such an extent as to endanger life, by its being inspired into the glottis, and putting an immediate stop to respiration." We by no means question the fact of such elongation occurring, but no *proof* is given by the author that death has ever been induced in the manner alleged. Mr. Thompson does not increase the confidence of the critical reader in his accuracy, by indulging in such loose and questionable statements, as that "elongation of the uvula occasionally destroys life in typhus fever, by producing almost instant suffocation."

The secretaries seem to have forgotten their critical functions, in allowing this paper to pass muster in its present state. The meaning of such sentences as the following—there are not a few such—may not be doubtful, but assuredly the language is not passable. "In the first case a somewhat hasty *examination had nearly lost a valuable life;* for had the paroxysm occurred in the night, or had *he* not been capable of great exertion, death would have certainly taken place."

VI. *A Report on Midwifery in Private Practice.* By Mr. C. B. Rose, of Swaffham.— Mr. Rose contributes a brief but really interesting statistical record of his obstetric practice during a series of years. He very correctly observes that, as reports of this kind have hitherto been chiefly furnished from the documents of public institutions, our knowledge on the subject of the statistics of parturition is in truth only applicable to the lower orders of the community. We trust the example he has so creditably shown them, will be followed by such members of the association as are engaged extensively in midwifery practice: in this way a mass of facts, from which most useful inferences respecting the value of female life might be drawn, would ere long be produced. Mr. Rose's cases, and the peculiarities attending them, are grouped in the following table:

| 600 Cases. | | Sex. | | Twins. | Preternatural Presentations. | | | Face. | Funis. | Hemorrhage. | | Convulsions. | Stillborn. |
|---|---|---|---|---|---|---|---|---|---|---|---|---|---|
| Nat. | Pret. | Boys. | Girls. | | Foot or knees. | Breech | Arm or elbow. | | | Unav. | Accid. | | |
| 582 | 18 | 316 | 294 | 10 | 7 | 9 | 2 | 1 | 2 | 2 | 18 | 2, and 2 with prem. sym. | 34 |

This practitioner met with one case in which the placenta and fœtal head were expelled together, the former having been pushed into the vagina by the latter; the hemorrhage was not alarming, and both mother and child did well.

The paper contains some not injudicious remarks on the exhibition of the ergot of rye in labour, which may be profitably consulted.

VII. *Cases of Hernia Cerebri, with Remarks.* By Mr. G. Mallett, of Bolton.—Mr. Mallett resides in a mining district, and has *consequently* met with several cases of severe fracture of the skull, and briefly relates the chief circumstances of five of these, four of them attended with encephalocele. We shall abridge the most remarkable of the latter. A circular portion of bone belonging to the frontal and parietal bones, and about one inch and a half in diameter, was driven by a falling piece of coal at least three quarters of an inch into the brain of a boy aged twelve years. When seen after the accident, the patient was sensible, the right side paralysed, vision of the right eye nearly gone, pupil dilated; there was neither fever nor delirium. After the lapse of a week a portion of brain protruded, gradually increasing in size until it measured six inches in its antero-posterior, three and a half in its transverse, and two inches in its vertical diameter. The mass was then removed with the knife, the patient being scarcely conscious of the passage of the instrument through its substance: there was a piece of bone found in its interior. In eleven weeks after the operation, the patient was able to walk a distance of upwards of two miles. Subsequently he recovered his original share of intelligence, and with the exception of persistent paralysis of the right arm, appeared in every respect as before the accident.

Mr. Mallett is of opinion that, inasmuch as in the four instances in which cerebral hernia occurred, a foreign body was discovered in the tissue of the brain; and in the case of fracture, uncomplicated by such protrusion, no fragment of bone or other extraneous matter was therein detected, encephalocele is, in the majority of cases, the direct result of foreign irritation.

In all the cases of protrusion, moderate pressure, and various topical applications, were first had recourse to, but in two were found totally ineffectual. Here the operation was performed. If, after ablation of the protruding substance, there should be a tendency to its reproduction, Mr. Mallett advises the employment of moderate pressure, and the daily use of a nitric acid lotion, containing forty drops of the acid to two ounces of water.

VIII. *A Case of Intussusceptio, with Separation of Five Inches of Intestine, followed by complete recovery.* By Mr. John Fox, of Cerne Abbas.—An interesting case, in which inflation of the bowels was had recourse to, and, though so late as the sixth day, with manifest advantage.

IX. *A Case of Molluscum.* By Mr. C. Fowler, of Cheltenham.—Correct descriptions of this singular affection are so rare, that we willingly transfer the following history (slightly abridged) to our pages:

"This singular affection first made its appearance about twenty or thirty years since, [the patient is a male, aged sixty-eight,] commencing in small accuminated elevations upon the waist and wrists, and gradually increasing to their present magnitude. The large and pendulous tumour hanging from the crest of the left ilium, and which it was customary with him to place in his breeches-pocket for support, whilst at work, was the first that made its appearance; this, and another large one between the arm and thorax of the same side, were the only ones which, from occasional subjection to pressure, gave him any degree of uneasiness or pain. These tumours were thickly disseminated over the whole cutaneous surfaces, from the crown of the head to the sole of the foot, and varied in size, from the smallest visible elevation of the skin to one on the left waist, which weighed about three quarters of a pound; some of them are attached by broad bases, and others by stems of various degrees of length and magnitude. The tumours upon the back and waist were the most thickly set of any, and looked like clusters of reddish grapes; those upon the abdomen and lips were longer and more irregular in form. The skin covering them was perfectly sound, but possessing more of a rose tint than the surrounding integument; in those, however that had been subjected to pressure upon their summits, the colour had changed to a dirty bluish gray; the skin, likewise, was very much attenuated, and easily raised from and rolled over the subjacent tumours; forcible compression with the fingers seemed to produce very little uneasiness in them, and the sensation to the touch, when handled, very nearly resembled that of fatty tumours, only somewhat harder in consistence, and many of them, particularly the larger ones, were distinctly lobulated. Two of moderate size, situated upon the abdomen, were removed, and on making a section of them, no very great difference was observable in them from the appearance of fatty tumours generally; there was the same semi-transparent yellowish tinge pervading the whole mass, as in the latter, but the consistence was denser, the cut surface smoother and firmer, and the substance more uniform throughout. The skin was loosely connected with them, and a fine cellular membrane surrounded the whole, and dipped into the interstices of those that were lobulated. The excision of these tumours was accompanied with the loss of but a few drops of blood." (p. 365.)

The case is illustrated by an excellent engraving.

X. *On the Physiology of the Brain as the Organ of the Mind.* By Dr. Cowan, of Reading.—We have noticed, in our last article, at considerable length, the subject of this interesting and important paper. Dr. Cowan is a philosophical, in other words, a reasonable phrenologist.

XI. *Remarks on the Use of the Fucus Esculentus as a Bougie.* By Dr. T. A. Aitkin, of Poole.—Dr. Aitkin has employed the stem of this plant, as a bougie, in several cases of stricture of the rectum, with uniform satisfaction to himself and his patients.

XII. *Observations on the Physiology and Function of the Liver.* By Dr. M'Cabe, of Cheltenham.—The drift of this communication is not very obvious; at any rate, it contains nothing worthy of notice, and its place might have been well supplied by matter of a more practical character.

XIII. XIV.—*Reports of the Town Infirmary and of the Asylum for Children at Birmingham.* By Mr. F. Ryland, of Birmingham.—These are very interesting papers, but furnish no materials for analysis. They deserve the attention of the medical topographer and statistician.

